# Impact of refractory and unexplained chronic cough on disease burden: a qualitative study

**DOI:** 10.1186/s12890-022-02171-z

**Published:** 2022-10-01

**Authors:** Naoya Ueda, Anzu Yakushiji, Jonathan Schelfhout, Shigeru Tokita, Takekazu Kubo

**Affiliations:** 1 Medical Affairs, MSD K.K., Tokyo, Japan; 2 IQVIA Solutions Japan K.K., Tokyo, Japan; 3 Merck & Co Inc, Rahway, NJ USA

**Keywords:** Chronic cough, Patient burden, Qualitative research, Cough variant asthma, Social communication

## Abstract

**Background:**

Chronic cough lasting for > 8 weeks is a common medical condition that burdens patients. This study aimed to qualitatively describe knowledge, awareness, experiences, and subtypes of burdens (physical, social, psychological) among Japanese patients with refractory chronic cough (refractory to treatment of underlying relevant medical conditions) and unexplained chronic cough (symptoms of unexplained origin).

**Methods:**

This non-interventional, cross-sectional study was conducted between February and March 2021 among patients (aged ≥ 20 years) with self-reported refractory or unexplained chronic cough. Subjects with a history of comorbid respiratory conditions were excluded. Eligible subjects participated in a 60-min online semi-structured interview. Verbatim terms from interviews were qualitatively transcribed and generated into word clouds, followed by a clustering analysis in which meaningful clusters were chosen, manually coded, and utterances and burdens categorized.

**Results:**

A total of 21 participants (95.2% with refractory chronic cough, mean age 53.5 years, and 76.2% being males) with Leicester Cough Questionnaire mean ± standard deviation scores of physical 4.8 ± 1.1, psychological 4.4 ± 1.3, social 4.9 ± 1.4, and total 14.1 ± 3.5 were included. The word cloud identified the most frequently used word (‘cough’); etiology (‘asthma’); and words associated with change in states (‘influence,’ ‘changing,’ ‘change’) and expressions (‘tough,’ ‘pain,’ ‘hard,’ ‘terrible,’ ‘unpleasant’). The patients experienced ‘mental/social burden,’ ‘physical burden,’ ‘impact on sleep and meals,’ ‘impact on work and housework,’ ‘impact on communication,’ ‘impact on hobbies and leisure,’ and ‘economic burden.’ By closed coding analysis, the situations or types of burden patients experienced from the cough were ordered sequentially as emotion, working style, acquaintanceship, hobbies and leisure, and sleeping pattern.

**Conclusions:**

The present study indicated that there were two types of participant clusters, in which one showed mainly the burdens in the social communications such as work-related communication and another one showed the burdens of relationships with others. Also, some participants highlighted ‘mental burden,’ on social life due to the current pandemic. To relieve these burdens, disease awareness and knowledge should be improved for patients with refractory and unexplained chronic cough.

*Trial registration* The trial was registered under UMIN-CTR as UMIN000042772, on 17/12/2020. The study was approved by the Medical Corporation Toukeikai Kitamachi Clinic (IRB registration number: 11001110).

**Supplementary Information:**

The online version contains supplementary material available at 10.1186/s12890-022-02171-z.

## Background

Cough is one of the most common symptoms experienced worldwide by patients seeking medical care [1]. It is one of the most frequently reported symptoms, and is responsible for up to 12% of overall consultations at outpatient clinics and hospitals in Japan [2, 3]. Cough is categorized based on duration: acute cough, subacute cough, and chronic cough, with each defined as lasting up to 3 weeks, 3 to 8 weeks, and lasting for more than 8 weeks, respectively [4, 5]. The global prevalence of chronic cough varies from 2 to 18% in different geographical regions, as identified in a systematic review and meta-analysis of 90 studies [6]. According to a recent online survey in Japan, the nationwide prevalence of chronic cough was 2.9% at the time of the survey, while 4.3% of the participants who experienced cough in the past 12 months had cough lasting more than 8 weeks [7]. In another internet-based survey of 1000 cough patients in Japan, chronic cough was reported in 23.2% of subjects who had cough at the time of the survey [8].

As a wide range of diseases may be associated with chronic cough in adults, the 2019 Japanese guidelines [9] recommend first exploring whether there is a single or major cause of cough, including respiratory infections, lung cancer, asthma, and chronic obstructive pulmonary disease (COPD). When the cause of chronic cough cannot be easily identified by medical interviews, physical findings, and chest X-rays, cough-variant asthma, atopic cough, gastroesophageal reflux disease (GERD), sinobronchial syndrome (SBS), and postinfectious cough are thought to be frequent underlying diseases [9]. A small proportion of chronic cough patients have persistent cough despite thorough investigations and treatment. In patients with refractory chronic cough, cough symptoms persist even after appropriate treatment of underlying medical condition in accordance with guidelines [10]. A patient may also be considered as having unexplained chronic cough, where no diagnosable cause for cough can be identified despite extensive evaluation of all possible causes [9, 11].

Currently, there are no specific treatments approved by the United States Food and Drug Administration, the European Medicines Agency, and Japanese regulatory agency (the Pharmaceuticals and Medical Devices Agency) for the treatment of refractory and unexplained chronic cough, and guidelines focus more on the treatment of underlying disease(s) [4, 12–14]. Diagnostic treatment for possible underlying disease of chronic cough is commonly practiced if the causes of cough cannot be easily identified [15]. In addition to treatments for underlying diseases, antitussives, including both narcotic and non-narcotic drugs, are frequently used despite concerns regarding their adverse effects and efficacy [9, 16]. Physiological and speech and language therapy intervention (PSLTI) and neuromodulators including gabapentin, amitriptyline, and pregabalin are also stated as therapeutic options for unexplained chronic cough and cough hypersensitivity syndrome in the Japanese guideline [9]. However, neuromodulators are off-label drugs for chronic cough in Japan and their side effects should be considered. Furthermore, PSLTI is rarely conducted in Japanese clinical settings.

A crucial aspect of chronic coughing is the burden it places on daily living. Patients with chronic cough experience extensive physical issues—including sleep disturbance and dysfunctional breathing [17–19], psychological issues such as anxiety and depression [20], social issues—including social isolation, limited daily life activities, and giving up hobbies, sports, and recreation activities [17], and increased economic burden—including direct and indirect medical costs, leading to a lower quality of life (QoL) [21]. Several studies have documented the influence of chronic cough on patients’ QoL. A small prospective study conducted in the United States identified a correlation between symptom duration and sickness impacting psychological and physical wellbeing [22]. A European study showed that 96% of patients with chronic cough had a change in their QoL, 70% of patients had an unsatisfactory experience with their doctors, and > 90% of patients felt that chronic cough had impacted their families [23]. Moreover, the burden of chronic cough is often long lasting and compounded by persistent symptoms despite numerous doctor visits, empirical treatment trials, and frequent medical testing [11]. In addition, the coronavirus disease 2019 (COVID-19) pandemic has led to overlap of chronic cough signs and symptoms, further exacerbating psychological stress in patients [24].

Although numerous studies on chronic cough have been conducted, knowledge and awareness on the topic are limited and very little information is available regarding the experiences and burden of chronic cough among the Japanese population. A significant proportion of patients with chronic cough are refractory to existing treatments. Qualitative data clarifying the impact of the disease and its burden is needed to improve patient care. Qualitative research is a valuable tool for exploring patient burden as it is interpretive and conducted via interviews, capturing the patient’s unique experiences related to their disease and its burden [25].

The aim of this study was to describe the experiences and burden of disease in patients with refractory and unexplained chronic cough in Japan using qualitative survey methods. In addition, the present study was also planned to describe the subtypes of burden, such as social, physical, and psychological burden, in patients with refractory and unexplained chronic cough in Japan.

## Methods

### Study design

This non-interventional, cross-sectional survey was conducted between February and March 2021 among patients who reported experiencing refractory or unexplained chronic cough. A 60-min online interview was conducted with each patient using a semi-structured interview guide (Additional file 1: Table S1). Information collected during the interviews was collated to qualitatively describe patient burden and experiences in relation to chronic cough.

### Participants

Participants were recruited through the patient panel of Rakuten research, consisting of a pool of approximately 2.3 million registered individuals residing in Japan [26]. The subjects were asked screening questions through the Rakuten panel followed by an online screening survey to assess their eligibility for enrollment in the study.

Adult subjects (≥ 20 years of age) with either refractory or unexplained chronic cough of duration > 8 weeks were eligible to participate in the study. Participants with refractory chronic cough were defined as those who had self-reported to have received treatment for asthma/cough-variant asthma, atopic cough/laryngeal allergy, GERD, postinfectious cough, or SBS based on a clinical diagnosis but whose cough persisted despite these treatments. Participants with unexplained chronic cough were defined as those who had chronic cough of unexplained origin even after clinical evaluation of possible underlying diseases. Subjects with immunocompromised status and respiratory diseases such as lung cancer, cystic fibrosis, idiopathic pulmonary fibrosis, chronic bronchitis, and moderate to severe COPD were excluded from the study. Considering the nature of the present study using qualitative interviews, participants with mental health issues such as schizophrenia and bipolar disorder that may confound the study results based on the investigators’ opinion were also excluded. Moreover, subjects who have a history of smoking which caused to exacerbate cough symptoms, those using a ventilator, those with intubation or tracheostomy, and those on angiotensin-converting enzyme inhibitors (ACEi), a recognized medication known to induce cough [27], were also excluded. Moreover, subjects with a history of substance abuse (within 1 year) were also excluded, as it might have worsened cough symptoms and confounded interview results. Subjects with suspected or confirmed COVID-19 diagnosis, who had fever, used any investigational drug within 30 days, or had difficulty communicating via online interview tools, were also excluded from the study. All eligible participants were asked to provide written informed consent for the 60-min interview.

Eligible participants were directed to complete the online preliminary survey, wherein information on demographic characteristics (age, gender, body mass index, etc.) and background characteristics (medical, smoking history, potential underlying disease diagnosis, etc.) was collected. Descriptive data on cough symptoms and patient reported outcome measures were also collected, including cough duration, duration of medical care, the Leicester Cough Questionnaire (LCQ), and a cough severity visual analog scale (VAS).

### Survey construction, validation, and administration

In order to develop a semi-structured interview guide, an extensive literature review was performed on recently published guidelines and articles on the burden of respiratory diseases, such as asthma and COPD. The concepts and variables covered in the study were based on the literature review and are described in detail in Table 1. In the current study, the final selection and exact wording of questions were determined with input from subjects with refractory chronic cough and unexplained chronic cough. The literature review and pilot interviews identified 7 constructs ‘physical burden,’ ‘mental and social burden,’ ‘impact on sleep and meals,’ ‘impact on work and housework,’ ‘impact on communication,’ ‘impact on hobbies and leisure,’ and ‘economic burden.’ Before each qualitative interview, participants were asked to rank the impact of each of the 7 constructs. The interviews were conducted in Japanese, which made participants comfortable and eliminated any potential language barriers. In every interview, the participant’s candid personal insights on each theme were collected.

**Table 1:** Concepts and variables covered

Variable	Concepts
Physical burden	Motion including change in the range of motion
	Movement including walking and use of public transport
	Behavior including ability of multitasking, concentration and change in the number of mistakes
Mental and social burden	Emotions including amount of frustration, emotional instability and pessimistic emotions
	Social interactions including change in the amount of socialization with friends, emotion towards family members
Impact on sleep and meals	Sleep including experience of insomnia and daytime sleepiness
	Eating including change in appetite and intake of certain types of food
Impact on work and housework	Employment including change in work efficiency, employment status
	Daily housework including cleaning, laundry
Impact on communication	Communication including oral and written communication
Impact on hobbies and leisure	Hobbies and leisure including changes in time spent for hobbies and entertainment
Economic burden	Economic burden including burden of treatment cost, and accessibility to medical institutions
Symptoms of cough	Severity of cough
	Influence of environment, season, and time of day on cough
	Healthcare resource use due to cough including experience of hospitalization and amount of hospital visits per month
Treatment	Treatment experiences
	Expectations for new treatment

### Statistical methods

Demographic and general health characteristics of patients were presented as mean ± standard deviation (SD), and data with non-normal distributions were presented as median (range).

**Fig. 1:**
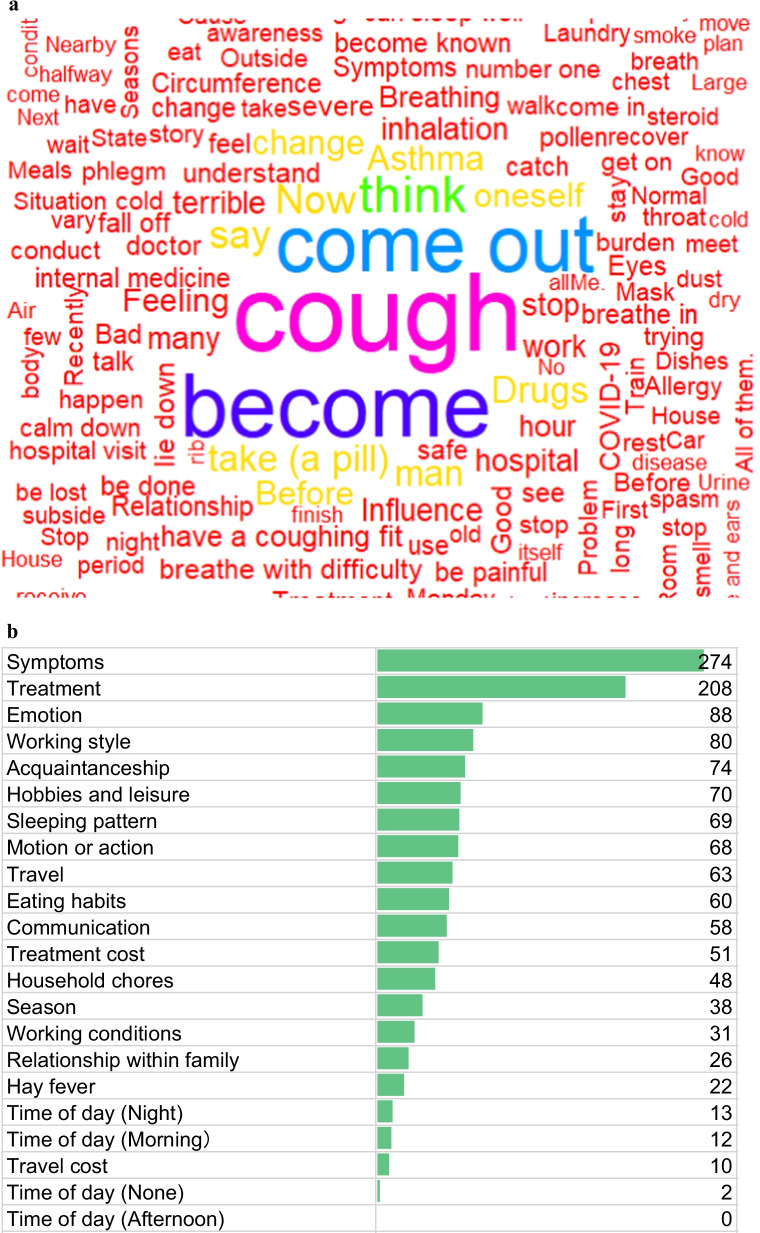
**a** Word cloud. **b** Number and classification of patient utterances

For qualitative data analysis, verbatim terms were transcribed and imported into software for analysis (R software version 4.0.5 and ATLAS.ti version 9 [28, 29]). The investigators created a verbatim transcript of recordings. Nonverbals used in the interviews, gestures made by patients, and the tone of their voice were also noted. With open coding, the investigators read through each note, line by line, and made a list of words and a network of words. Open coding with word clouds and thematic network analysis was carried out followed by a clustering analysis using R software. This technique aided in the classification of the variable data by grouping objects into individual classes. The strength of clustering analysis is that it does not require representative data; as it suggests ordering of the available data, it adequately analyzes smaller qualitative samples (Fig. 1a).

A cluster dendrogram was plotted from all the transcripts in the study (see Additional file 1: Fig. S1 for the cluster dendrogram). The dendrogram consists of stacked branches (called clades) and illustrates the arrangement of clusters produced by the corresponding analyses. The main branches break down into smaller branches to represent further data received from each patient interview. The arrangement of the clades reveals how similar they are to each other (e.g., 2 leaves in the same clade are more similar than 2 leaves in another clade). The Y-axis (height of the branch) shows how close data points or clusters are from one another. The taller the branch, the further and more different the clusters were assumed to be.

After identification of words and a network of words, closed coding was performed to identify pre-established items using a specialized qualitative analysis software program (ATLAS.ti). Coding for all transcripts were done by two coders, with a concordance rate of 93.2%. Once the coding was completed, a discussion was held to reach an agreement on the final list of codes. The network of words was narrowed down to 200 words by the frequency of use, codes defined, and a final code list was generated. In order to perform a clustering analysis, data were manipulated to make them suitable for the clustering analysis software; a clustering technique was chosen and measured similarity; and a meaningful number of clusters was then chosen for analysis. The quotes of participants (utterances) expressing their burden of cough were manually coded and categorized according to codes developed based on the domains of interest.

The sample size was calculated based on the Turner-Bowker qualitative interview saturation point method [30]. According to the method above, 97% of total concepts emerged by the 20th qualitative interview. Therefore, we speculated that this study aimed to reach a saturation point of 95% by recruiting and conducting at least 20 interviews.

## Results

### Participants’ characteristics

#### Patients’ demographic characteristics

Twenty-one patients with a self-reported diagnosis of either refractory or unexplained chronic cough participated in the study interviews. The demographic details and clinical characteristics of participants are presented in Table 2. The mean (SD) age of participants was 53.5 (11.9) years, and the majority (61.9%) were between 50 and 70 years. The 21 participants consisted of 16 men and 5 women (76.2% and 23.8%, respectively). In terms of smoking history, 17 of participants (81.0%) had no smoking history, whereas the rest were past smokers (19.0%). The majority of the participants were full-time workers (66.7%).

**Table 2:** Patient characteristics in Interviews

	n	%
*Gender*		
Male	16	76.2%
Female	5	23.8%
*Age (years)*		
30–39	4	19.0%
40–49	3	14.3%
50–59	7	33.3%
60–69	6	28.6%
≥ 70	1	4.8%
Mean (SD)	53.5 (11.9)	
*Working status*		
Full-time	14	66.7%
Stay-at-home spouses (or partners)	2	9.5%
Retired	2	9.5%
Other	3	14.3%
*Duration of cough symptoms*		
1 year or more	20	95.2%
2–4 months	1	4.8%
*Duration of medical care of cough*		
1 year or more	20	95.2%
1–2 months	1	4.8%
*Primary disease*		
Refractory	20	95.2%
Unexplained	1	4.8%
*Hypertension other than ACE*		
Yes	6	28.6%
*Family history of cough*		
Yes	1	4.8%
*Smoking status*		
I used to smoke, but I quit	4	19.0%
I've never smoked	17	81.0%
*BMI (kg/m*^*2*^*)*		
≤ 19	3	14.3%
20–24	11	52.4%
25–29	5	23.8%
≥ 30	2	9.5%
Mean (SD)	24.1(4.3)	
Underlying diseases*		
Asthma/Cough-variant asthma	20	95.2%
Atopic cough	2	9.5%
Postinfectious cough	1	4.8%
Undefined	1	4.8%
Other	1	4.8%
*Cough VAS (past 2 weeks)*		
0–29	8	38.1%
30–59	9	42.9%
60–100	4	19.0%
Mean (SD)	36.2 (21.6)	
*Cough VAS (worst day)*		
0–29	5	23.8%
30–59	9	42.9%
60–100	7	33.3%
Mean (SD)	46.7 (25.4)	
*LCQ (mean, SD)*		
Physical, mean (SD)	4.8 (1.1)	
Psychological, mean (SD)	4.4 (1.3)	
Social, mean (SD)	4.9 (1.4)	
Total, mean (SD)	14.1 (3.5)	

*BMI* body mass index; *LCQ* Leicester Cough Questionnaire; *SD* standard deviation; *VAS* visual analog scale

^*^Multiple answers could be provided for this category

#### Patients’ clinical characteristics

Of 21 participants, 20 participants (95.2%) were self-reported refractory chronic cough, and the rest (4.8%) was unexplained chronic cough. Most of participants (95.2%) had experienced symptoms and medical care of cough for 1 year or more. Only 1 participant (4.8%) had a family history of cough. Asthma/cough-variant asthma was the most common underlying disease (95.2%), as mentioned by all subjects with refractory chronic cough (n = 20). Atopic cough and postinfectious cough were also mentioned as underlying diseases by 9.5% and 4.8% of the participants, respectively. The mean score on the cough severity VAS was 36.2 druring the 2 weeks prior to the interview, and 46.7 on the worst day during these two weeks. The mean (SD) LCQ total score was 14.1 (3.5) with mean (SD) domain scores of 4.8 (1.1) for the physical domain, 4.4 (1.3) for the psychological domain, and 4.9 (1.4) for the social domain.

### Qualitative data analysis

#### Open coding

The first step in analyzing qualitative data involved transcribing video and audio recordings. Since the interviews were conducted in Japanese, the analysis was also subsequently conducted in Japanese.

The most frequent word that appeared during transcription of the interviews by open coding was ‘cough,’ and the word ‘asthma’ was sporadically used as an etiology (Fig. 1a). The open coding words associated with change in states were ‘influence,’ ‘changing,’ and ‘change.’ While the words associated with burden expressions were openly coded as ‘tough,’ ‘pain,’ ‘hard,’ ‘terrible,’ and ‘unpleasant.’

Once the words were identified by open coding, networks were created using combinations of identical words which occurred 4 or more times across all interviews. The words ‘understanding,’ ‘coping,’ and ‘cough’ were connected to the same network. Similarly, a series of words such as ‘concentration–fall,’ ‘thorny–throat,’ and ‘inhalation-medication-bronchi’ was connected. Based on these findings, the word-group relation with symptoms and the disease burden was observed. Once networks were developed, clusters were created to discover words and phrases related to the burden of a specific sense of personal experience with cough and relationships with others. The subjects were divided into 2 mutually exclusive clusters (see Additional file 1: Fig. S1). The first cluster demonstrated the expressions of a specific sense of social burden, based on subjects’ personal experience with cough. The burden was identified from interview responses, such as thoughts of canceling their schedule or quitting their job due to work-related issues and delaying or canceling a leisure-related activity. Participants also mentioned feelings of embarrassment when they could not communicate well due to coughing. During the interview, they selected either ‘work’ or ‘communication’ as the paramount burden in interdomain ranking.

In the second cluster, subjects spoke about the burden of relationships with others. During the interview, they selected mental, physical, sleep, and dietary issues as representative burdens of cough. The participant responses were indicative of their feelings of not intending to trouble others and not wanting to receive attention from others because of their cough. The subjects felt embarrassed when coughing in public, and they were also afraid of talking to people because they were concerned that if they talked, their coughing would start. Some participants mentioned that they were apprehensive about taking their medication each time they coughed. Some participants mentioned that they were worried about coughing even while wearing a mask, and afraid that people around them would suspect their physical condition as being related to COVID-19.

#### Closed coding

The clustering analysis was followed by closed coding. Closed coding revealed the most frequently occurring code as ‘symptoms’ identified 274 times followed by ‘treatment’ identified 208 times. Within the ‘symptoms’ code, the three most frequently mentioned words were ‘asthma’, ‘hospital’ and ‘symptoms’, mentioned by 16 (76.2%), 10 (47.6%) and 10 (47.6%) participants, respectively. Within the ‘treatment’ code, the three most frequently mentioned words were ‘inhale’, ‘asthma’ and ‘hospital’, mentioned by 18 (85.7%), 13 (61.9%) and 11 (52.4%) participants, respectively. The number and classification of utterances are described in Fig. 1b. The situation or type of burden patients experienced from the cough were ordered sequentially as emotion, working style, acquaintanceship, hobbies and leisure, and sleeping pattern (Fig. 1b).

### Disease burden

The results of targeted literature review showed the impact of cough on different domains, such as impact on motion and body movement, concentration and behavior, emotion, sleeping and meals, work and housework, family, friends and communications, hobbies and leisure, economic burden, and the burden of hospital visitation. The descriptive quotes for each burden are presented in Table 3.

**Table 3:** Descriptive quotes for each burden

Type of burden	Descriptive quote
Impact on motion and body movement	Driving is scary. If I cough when I’m holding the wheel, I can’t see ahead. I don’t drive much now
	It’s a problem when there are people around me such as when I am on the train. People would think I have COVID-19 nowadays, or a cold during the pre-COVID era. In such cases, I would endure it or hold my mouth tightly and try to keep the sound as low as possible
	I started to wheeze just by going up the stairs at the station. It’s hard to walk in the station because I feel like I’m walking fast to keep up with the crowd. Going up and down the stairs is a big burden for me
	Coughing can cause a loss of concentration, momentary difficulty in breathing, or pain in my lungs
Impact on concentration and behavior	When I stop what I’m doing because of coughing, I get some rest. But I feel like that resets my thinking
Impact on emotion	Asthma makes me feel like I have lost. I’ve lost in life
	I am thinking why I got to this stage, it’s annoying, and I’m always worried about going out, and about what I would do if I couldn’t stop coughing again when I’m on a train or plane
Impact on sleeping and Meals	Recently, when I go to bed or wake up in the morning, I have a cough, so I get my inhaler and use it to stop my cough
	When I’m in bed and have a cough, I try to move to a place where I can be alone
	I get sleepy the next day. When it’s bad, I take a nap the afternoon of the next day
	Spicy food. Because it induces (my cough). When I start coughing due to the spiciness, I end up with an asthmatic cough, so I eat less spicy food (nowadays)
	Sometimes I choke while I’m eating, and choke when I am drinking, and cough even more. Frankly speaking, I am annoyed when it comes to food
Impact on work and housework	Coughing often interrupts my work
	It becomes a nuisance to work. I feel like I don’t want to work anymore
	Regarding cars, cough doesn’t generally restrict me on the job, but it does prevent me from working outside of the office. This stopped me from pursuing my career plan
	I have left early several times, but I never take an entire day off. I have to leave early because I have a bad cough, or I have to go to the hospital… only by about 30 min to an hour
	I’m taking a leave of absence on company orders. When it began, I was suffering from the same difficulty in breathing, my daily activities were limited, and I had trouble conversing
	Laundry usually requires vertical motion of the body. Lifting up clothing to dry them. It was so hard that I had to ask someone else to do it
	Sometimes I can’t go shopping or cook if it’s too bad
Impact on family, friends, and communications	My boss would sometimes say, ‘You have to get well’ or ‘coughing is annoying for others.’
	People often ask, ‘Are you okay?’ but obviously I’m not all right at all
	Both my parents and wife know about it (my cough), so it doesn’t affect us much
	I feel sorry for making others wait when I physically can’t speak
Impact on hobbies and leisure	I sing in opera, so I’m basically finished if I cough. I usually don’t cough while performing due to nervousness, but I have difficulty controlling my breathing during practice. However, I don’t cough fully. I increase my inhalant beforehand
Impact on economic burden and burden on hospital visit	The medicine is expensive. I take a lot of it. It has become an economic burden
	(The hospital) is within walking distance…so, I don’t feel much burden because of having to visit the hospital
	Traveling is a burden, as the hospital is far away; it costs more than 1000 yen for each trip, in addition to the medication cost it costs more than 6000–7000 yen per month
	Money isn’t a burden for me because I don’t go to doctors that I have to wait for. Time is more of a burden
Symptoms and treatment of cough	A dry cough. It troubles me almost every day
	When I start coughing, it’s a cough that makes a rattling sound inside me, unlike a normal cough
	I also have hay fever, I don’t know if that’s the reason, but I always sneeze when I cough and feel a little unwell in early spring
	At the turn of the season, I feel so sluggish that I just want to sit down
	When I get up in the morning. I feel like phlegm is stuck in my throat, which is the worst feeling
	I have a long continual cough in the middle of the night or in the morning. During the day, it repeatedly comes and goes
	My current medication is fine. I strongly feel the side effects of taking new treatments, so I wonder…
	I don’t want to take any medicine if possible. Medicines are not natural, so I feel like they are not good for me
	I’ve had times before when I was introduced to new medication or told that I need to change my medication, so I have no hesitation to do so
	I would like to try (new medication). If I was asked to be a human guinea pig for a clinical trial, I would

#### Impact on motion, action, and travel

In terms of impact on motion and body movement, 11 participants (52.4%) mentioned they had pain and shortness of breath resulting in restricted body movement. Ultimately, the participants’ movement and range of motion were adversely impacted. Specifically, 7 participants (33.3%) mentioned difficulties with going up and down the stairs and 9 (42.9%) mentioned walking difficulties. Fifteen participants (71.4%) mentioned the burden of using various forms of transportation, especially due to COVID-19 with participants suppressing the urge to cough while using public transportation, to avoid suspicion of having COVID-19. In 6 cases (28.6%), coughing adversely impacted driving, by forcing the driving participant to stop the car when coughing, and in extreme cases, stop driving completely, as coughing impacted their vision and ability to drive safely.

#### Impact on concentration and behavior

The participants experienced behavioral changes due to persistent coughing. Fifteen participants (71.4%) complained of reduced productivity due to poor concentration, resulting in the inability to achieve their goals. Five participants (23.8%) indicated that they started to have negative feelings towards responsibilities due to cough interruptions such as, avoiding initiating tasks at all, out of fear of making more mistakes. Eight participants (38.1%) also felt reluctant about and avoided going outside unnecessarily.

#### Impact on emotions

Eighteen participants (85.7%) said they had feelings of anger, negative feelings, such as feeling guilty, and a sense of sadness and anxiety, due to long-term coughing. Pessimism related to the future was observed in the interviews, consequently leading to patients not being motivated to try new things.

#### Impact on sleeping and meals

The interviews indicated difficulties with breathing and chest pain, which resulted in trouble sleeping at night. Seventeen participants (81.0%) mentioned having trouble sleeping at night, and 13 participants (61.9%) noted that cough sometimes prevented them from sleeping all night, affecting their work efficiency the next day. Temporal effects such as coughing at dawn or midnight were confirmed. Nine participants (42.9%) mentioned about coping methods to fall asleep, e.g., when their symptoms were bad, sitting on the sofa would help them fall asleep, because lying down caused them to cough. Five participants (23.8%) had mentioned the effect that their cough had on other people. They would sleep in separate bedrooms or hold-in their cough as not to bother others. Seventeen participants (81.0%) experienced coughing and choking triggered by eating specific types of foods, including steaming dishes, noodles, and spicy foods. They were concerned coughing made others uncomfortable. Thirteen participants (61.9%) mentioned that they had increased their water intake to prevent coughing.

#### Impact on work and housework

Coughing sometimes interrupted the participants’ work and made them lose their motivation. Ten participants (47.6%) had difficulties with work involving social interactions such as phone calls, presentations, and counter business due to sudden coughing. As a result, chronic cough triggered a pronounced psychological burden, as it was a concern for the people around them. Coughing had serious adverse implications on work for 11 participants (52.4%), such as restrictions on car use, forced change of job, and leave of absence. It was shown that participants often tried to take medications to prevent coughing when dealing with clients or attending meetings. Eleven participants (52.4%) had to explain the nature of their cough to colleagues and clients, so as not to mislead them into thinking they had an infection. Housework burdens related to managing humidity, such as cleaning the bathroom, were highlighted by the participants. Eight patients (38.1%) mentioned that they required a break due to their cough. They reported using various approaches, including utilizing humidifiers when doing housework, to mitigate the effects of cough on their daily activities.

#### Impact on family, friends, and communications

The participants were interviewed regarding the impact of chronic cough on family, friends, and communications. Due to coughing, 14 patients (66.7%) spoke about how people around them would get worried and in some instances advise them to see a cough specialist while providing possible reasons for their cough, such as their history of asthma. Six participants (28.6%) indicated that they were sympathetic more often about other people with cough. The participants mentioned that gaining a better understanding from the people around them decreased the burden on them. Family members of the patients were often worried and supportive. Sixteen participants (76.2%) mentioned that coughing often interrupted conversations, including phone calls. It was highlighted that in 14 participants (66.7%), although the interruptions had not yet affected their relationships with others, they were concerned about how they felt about their coughing and felt guilty. Moreover, due to coughing, the participants ended conversations quickly and felt the direct burden of others feeling offended.

#### Impact on hobbies and leisure

Fifteen participants (71.4%) commented on how they had to quit or reduce the time spent on hobbies and leisure activities due to their cough. Four participants (19.0%) expressed that while the time spent pursuing hobbies had not changed, they were forced to choose timeframes and transportation means that decreased interaction with others. Four participants also mentioned the anxiety associated with their cough led to them ensuring that their medications and cough suppressant candies were always readily available.

#### Economic burden and burden of hospital visits

When the participants were interviewed about the economic burden and burden of hospital visits, 15 participants (71.4%) mentioned that medical expenses including the medications they took were expensive. One participant mentioned that their medicine cost burden was increasing: their medications cost around $74 (\10,000 exchange rate based on rates from Bank of Japan, date: June 28th, 2022) per month, which concerned them. Twelve participants (57.1%) mentioned time lost for hospital visits were a burden. The hospital was not easily accessible for 7 participants (33.3%), leading to increased expenditure of time and money on hospital visits. Other burdens highlighted by the participants included the long waiting times at the hospital. On the other hand, other participants felt less burdened in this regard, as the hospital was close to their workplace or residence.

#### Impact on symptoms and treatment of cough

Eleven participants (52.4%) experienced chest pain while coughing, and 17 participants (81.0%) had trouble breathing. Seasonal influences and diurnal time variation in cough symptoms were noticed by 19 participants (90.5%), based on their remarks during the interviews. The use of an inhaler as a symptomatic or prophylactic treatment was highlighted by 17 patients (81.0%), and use of oral medications were mentioned by 17 patients (81.0%). Thirteen participants (61.9%) felt that their symptoms were largely controlled by their current medications, and 11 participants (52.4%) were not satisfied, as the disease had not been fully cured. Twelve participants (57.1%) were reluctant to change their medications; even so, 16 participants (76.2%) wanted a new, more effective medication for their problem.

## Discussion

Although patients with chronic cough are known to have significantly poor QoL [19], the present narrative study revealed specific and qualitative burdens on patients due to chronic cough in Japan. The mean LCQ scores for the physical, psychological, and social domains (4.8, 4.4, and 4.9, respectively) in the present study were consistent with those from a previous online survey in Japan [19]. The study aimed at evaluating the experiences of patients and the burden of refractory or unexplained chronic cough using qualitative interviews, which has not been extensively researched in the past.

The majority of participants (95.2%) had refractory chronic cough, and only 1 participant (4.8%) was identified to have unexplained chronic cough. In accordance with previously reported studies, 95.2% of the participants in this study had a duration of cough symptoms of more than a year with asthma/cough-variant asthma as a presumptive cause [8, 31]. Unlike in previous studies, a male preponderance was noted in this study [32, 33].

The qualitative study could illustrate individual patient burden due to refractory and unexplained chronic cough in detail by independently collecting each patient narrative. Text analysis creates consistent and accurate data that is nonbiased and can be utilized to interpret the transcription of the interviews. The generated cluster and network of words helped to identify the relationship between disease burden and the participants’ experiences and social situations. Several studies reported that there was a gap in perception between physicians and patients in regard to the impact of symptoms on daily life [34, 35]. It may also help clinicians in evaluating the best treatment plan for patients with chronic cough; this will also help clinicians to understand the need to reduce disease burden, along with treatment of symptoms.

In the current study, physical burden, such as pain in chest, shortness of breath, and coughing, prevented subjects from falling asleep. In addition, insomnia was noted as affecting work the following day. Observations related to sleeping pattern changes due to coughing were recorded, such as ‘*I get sleepy the next day*,’ ‘*When it’s bad, I take a nap in the afternoon of the next day*.’ Our literature search identified a close association between cough symptoms and the severity of insomnia in patients with COPD [36].

Social burden emerged as a pain point for most participants, as observed in terms of impact on family, friends, and ability to communicate with others as previously reported in the United Kingdom [33, 37]. The subjects indicated that uncontrollable coughing led to unwanted attention from others and the burden was exaggerated especially during the COVID-19 pandemic due to the similarity in symptoms of chronic cough and COVID-19.

A web-based survey in Japan evaluated the prevalence of psychological distress due to the COVID-19 pandemic among the general population, wherein the psychological burden was evident in 50.3% of males and 52.6% of females, as measured with the help of the Kessler 6 Psychological Distress Scale (> 5) [24].

In a study in Africa, health care costs for patients with chronic cough are found to be mainly influenced by the costs of transportation (mean: US$ 1.4) and drugs (mean: US$ 1.3), varying with the demographic and socioeconomic characteristics of patients [21]. Furthermore, a study in the United Kingdom found that higher healthcare costs (per person-year) were observed in patients with chronic cough compared to those with multiple or single events of acute cough [38]. Although the economic burden is dependent on the amount of medication prescribed, a minor economic burden was noted in this study for treatment and hospital visitation due to the robust Japanese insurance system providing enough coverage and the limited impact of coughing on employment status. Apart from the above, there were remarks suggesting the burden of time constraints for patients (e.g., travel time to the hospital by train, and long waiting times).

All the study participants received treatment for chronic cough symptoms mostly based on the diagnoses of their underlying diseases, but the majority of the patients were not satisfied with their treatment. There was 5 to 10% of treatment resistance in patients complaining of chronic cough, reaching 40% in higher medical institutions [39, 40]. According to the CHEST guideline, speech therapy and gabapentin were recommended for patients with unexplained chronic cough who are refractory to empirical therapies [41]. However, speech therapy is rarely offered in Japan because the expertise and skills of speech therapists and psychologists are not sufficient, and standardization of the therapy is not established [9]. Neuromodulators such as gabapentin are off-label for refractory and unexplained cough in Japan and evidence of the efficacy and safety for patients with refractory and unexplained chronic cough on neuromodulators are still not enough as stated in the Japanese guideline [9]. Since persistent cough impaired patients’ QoL for a long time, it is important to find an effective treatment approach for refractory and unexplained cough. If the participants’ symptoms were controlled by treatment, the potential burden of chronic cough had not yet surfaced or their burdens were attenuated in line with the previous studies on acute cough [22, 42]. Therefore, appropriate treatment for chronic cough that controls the symptom well could be one of the critical factors to reduce patient burden, which might improve patient QoL.

## Limitations

One potential limitation of the current study is a lack of generalizability due to the web-based recruitment used. Recruiting patients via the internet may result in lower representation of the elderly and others less likely to be identified via the internet [43].

In addition, the diagnoses of refractory and unexplained chronic cough, their underlying diseases, and diseases within exclusion criteria were all based on self-reporting, and it was difficult to check how exactly they reflect the actual medical records. Therefore, it cannot be denied that some respondents who were not intended as targets for recruitment, might have answered without knowing their exact diagnosis and health condition. Furthermore, there were no remarkable differences in the burden in patients with refractory and unexplained chronic cough in this survey, since the current study recruited only 1 patient with unexplained chronic cough. The burdens due to unexplained chronic cough needs further investigation in a larger population.

Based on experience in conducting qualitative research studies, attainment of adequate sample size depends on multiple factors and considerations—including quality of data, nature of concepts of interest, scope and complexity of research questions, homogeneity or heterogeneity of the sample, stratification and subgroups, and study methods used.

Further, future real-world studies are required to quantitatively evaluate whether the specific burden due to chronic cough identified in this study can be regarded as a more common burden in the population. Also, further clinical studies in Japan exploring the association between disease burden and treatment adherence in the context of chronic cough are warranted.

## Conclusion

This study evaluated the physical, social, and psychological burden in patients with refractory and unexplained chronic cough in Japan. The following domains of disease burden were identified: physical and mental burden, sleep and meals, work and housework, communication, hobbies and leisure, and economic burden. The social burden was shown to have a major impact (especially from the pressure and attention patients get from their surroundings), which might be exaggerated by the COVID-19 pandemic. In considering the therapeutic effect and value of treatments, it is necessary for the clinician to focus on patients’ chronic cough burden, especially in relation to the social aspect in Japan.

## Supplementary Information

**Additional file 1**. **Supplementary Figure 1** Clustering of patients from all transcripts. **Supplementary Table 2** Semi-structured interview guide for individual interviews for the patients.

## Data Availability

The datasets generated and analysed sets during the current study are not publicly available due to privacy protection and sensitivity issues. This study does not have ethics approval to share the participants’ narratives publicly. The datasets are not available to protect participant confidentiality. Regarding extracted data except personally identifiable data, the data may be available from the corresponding author upon reasonable request.

## Abbreviations

COPD: Chronic obstructive pulmonary disease
COVID-19: Coronavirus disease 2019
GERD: Gastroesophageal reflux disease
LCQ: Leicester Cough Questionnaire
SBS: Sinobronchial syndrome
SD: Standard deviation
QoL: Quality of life
VAS: Visual analog scale
