# Supplementary material for: Impact of refractory and unexplained chronic cough on disease burden: a qualitative study
Source: BMC Pulm Med. 2022 Oct 1;22:372. doi: 10.1186/s12890-022-02171-z (PMC9526196; doi:10.1186/s12890-022-02171-z)
Supplement: Supplementary file 1 — Additional file 1. Supplementary Figure 1 Clustering of patients from all transcripts. Supplementary Table 2 Semi-structured interview guide for individual interviews for the patients. [file 12890_2022_2171_MOESM1_ESM.pdf]

Additional file 1

Supplementary Figure 1 Clustering of patients from all transcripts

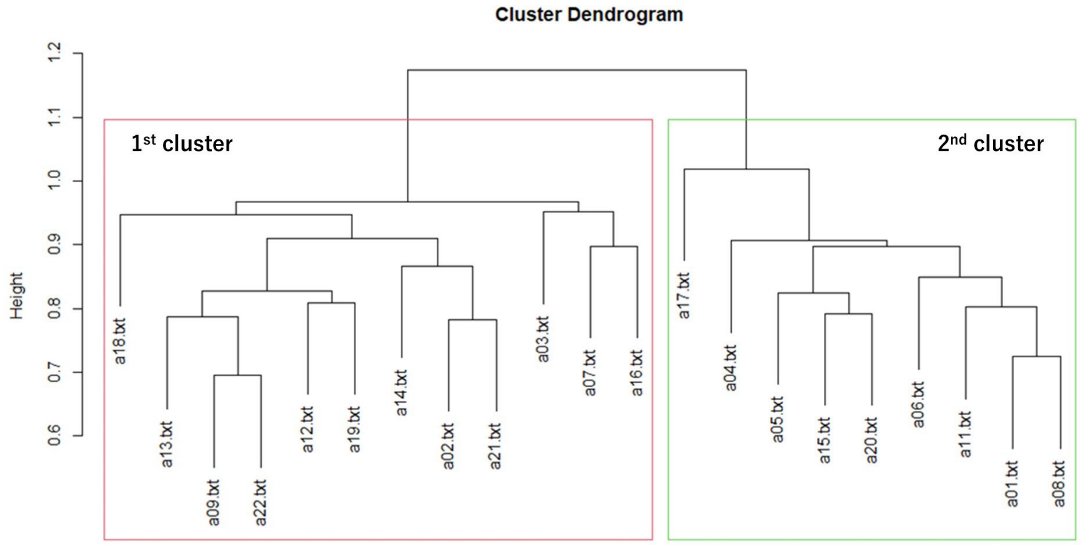

Supplementary Table 2 Semi-structured interview guide for individual interviews for the patients

Interview Related to Chronic Cough

Interview Guide*

Time	Item
3min/<br><br>3min.	[Participants were asked to return a signed informed consent form in advance.<br>The online preliminary survey wherein information on demographic characteristics (age, gender, body mass index , etc.) and background characteristics (medical, smoking history, potential underlying disease diagnosis, etc.) were conducted before the interview.]<br><br>Part 1. Explanation of Individual Interview/ Confirmation of Background Information<br>• Thank you for participating in today's interview.<br>• I am working for a company called IQVIA and will be taking the role of interviewer today.<br>• In this interview, I will not call you by your name during the interview to protect your personal information. Please make sure you do not share your full name.<br>• As you have been informed in advance, I would like to ask about the symptoms of your cough and its impacts on various situations. The purpose of this interview is to hear patient narratives, in order to reflect them in future development of cough treatments. Therefore, it would be greatly appreciated if you could tell us your feelings and thoughts on your cough symptoms.<br>• The interview will take 60 minutes. If you feel the urge to cough during the interview, you do not need to forcibly stop coughing. Also, if you feel unwell or uncomfortable, please let me know immediately.<br>• This interview is recorded, and the data collected will be analyzed. These recorded video and audio files will be used only for the purpose of analysis, we will not use it for any other purpose. This interview will also be observed by other project members such as analysts and personnel of the client company. However, your personal information is protected and will not be disclosed to anyone other than members of this project.<br>• As stated in the informed consent document, please confirm that, in the instance we hear about the occurrence of adverse effects of a specific drug during this interview, we are obligated to report it to the client pharmaceutical company.

Time	Item
	• Do you have any questions about the interview so far?<br><br>• Confirm screening information<br>➢ Family members living together<br>➢ Employment status, work status
17min/<br><br>20min.	Part 2. Symptoms and Treatment of Cough<br>• When did your cough symptoms start?<br>➢ What were the symptoms like at the beginning?<br>• When did you first consult a doctor for coughing?<br>• What did your physician tell you at the initial examination?<br>➢ What did your doctor explain to you regarding the cause of your cough?<br>• What type of medical institution are you visiting? (Clinic, neighborhood hospital, university hospital, etc.)<br>➢ Department: Has there been any changes in the type and number of departments you visit?<br>➢ Are you visiting a cough specialist or a general practitioner?<br>• How often do you visit your medical institution for coughing?<br>➢ Do you have a regular visit, or do you visit a medical institution only when your cough symptoms worsen?<br>• Have you been hospitalized due to your cough symptoms either directly or indirectly?<br><br>• What kind of treatments have you received so far?<br>[Do not ask specific names of drugs. Ask general questions such as whether it's OTC or Rx, or its duration.]<br><br>• What treatments are you currently taking?<br>➢ Dose frequency<br>➢ Type of medicine (inhaled or oral, etc.)<br>➢ Are you satisfied with the current treatment? How much have your symptoms improved since you started the current treatment?<br>• What are your expectations for future treatments?<br>➢ Do you have a desire to receive new treatment?<br><br>• What are your cough symptoms?<br>➢ How would you describe your cough? (e.g., dry cough or cough with sputum, etc.)<br>• How long do symptom(s) last once you cough?

<table>
<tr><th>Time</th><th>Item</th></tr>
<tr><td></td><td>
➢ Does your cough last continuously or intermittent? How long is the interval of your cough?
➢ How is your physical condition when coughing continuously? (e.g., dyspnea, pain, etc.)
➢ How do you feel while this is happening?
• How many days a week do you have cough symptoms?
• Have you ever been transported by ambulance due to your cough symptoms?

• Is there a specific time of day when your cough symptoms get better or worse? (e.g., morning, evening or mid-night)
• Do you feel that your environment, such as house dust and air pollution, impact your condition?
➢ How do you deal with environmental factors?
• Do you feel that the seasons or climate, such as dryness, humidity, and pollen, impact your cough symptoms?

• Do you have any diseases or symptoms other than cough?
• Do you have any disease or symptoms that could be caused by cough? For example, some people experience the following symptoms due to cough.

[Show the list as below for reference]
<table>
<tr><td>Insomnia</td></tr>
<tr><td>Depression</td></tr>
<tr><td>Syncope</td></tr>
<tr><td>Urinary incontinence</td></tr>
<tr><td>Chest pain / rib fracture</td></tr>
<tr><td>Headache</td></tr>
</table>
</td></tr>
</table>

Time	Item
40min/<br><br>60min.	Part 3. Burdens from cough<br>• What is the biggest issue or pain caused from cough symptoms? [Spontaneous answers only. Moderator do not thoroughly investigate in order to ask questions later.]<br><br>Now, I'd like to ask about how the following factors are influenced by your cough.<br><br>First, let me ask about its impact on your mind and body.<br>**[Impact on motion and body movement]**<br><br>• Has your range of motion changed since you began experiencing cough symptoms?<br>➢ Are there any motions that you are no longer able to do?<br>➢ How much do you think cough is impacting on your motion?<br>• Do you have any problems with movement? (e.g., easily feel tired or cannot move)<br>➢ Do you have any movement problems inside your room?<br>➢ Do you have any problems with movement inside buildings? (e.g., walking through the train station or building)<br>➢ Can you walk around the neighborhood? (e.g., walk to the nearby supermarket)<br>➢ Can you ride trains or buses? (e.g., any problem with standing inside)<br>• How many days a week do you perform strong physical activities? (e.g., carrying heavy luggage, biking up the slope, jogging, playing tennis singles)<br>➢ When you perform strong physical activities, how many hours do you exercise in total?<br>➢ During longer periods of time, are there any restrictions on the time spent on physical activities? (e.g., take rest the next day after going out)<br><br>• Do you take more breaks during activity?<br>• Do you have any problems with walking?<br>➢ Can you walk without a cane or a walker?<br>➢ Is the time you can keep walking the same as before?<br>➢ Can you walk up slopes?

Time	Item
	**[Impact on concentration and behavior]**<br><br>• Have you experienced any of the following changes since coughing started?<br>[Show the list and check for each factor.]<br><br>Lack of concentration<br>Making more mistakes than before when doing simple tasks<br>Lack of planning<br>Difficulty in multitasking<br>Taking more time to finish multiple tasks<br><br>**[Impact on emotions]**<br><br>• Have you experienced any changes with emotions since you began having coughing symptoms? What kind of changes?<br>[Spontaneous answer.]<br><br>• Have you felt any of the following changes since coughing started?<br>[Ask for each item]<br><br>Have you had calm and peaceful feelings?<br>Have you felt energetic?<br>Have you felt depressed?<br><br>➢ What do you think are the reasons behind these feelings?<br>[Skip if respondent cannot answer]<br><br>**[Impact on sleep and diet]**

Time	Item
	Now I'm going to ask about sleep.<br><br>• Are you able to sleep well despite having cough symptoms?<br>➢ Have you ever had trouble falling asleep due to cough?<br>• Do you feel sleepiness during daytime?<br><br>Now, I will ask about diet.<br>• Do you have an appetite?<br>➢ Has your daily amount of meals changed?<br>➢ How about your daily water intake?<br>➢ How about the amount of alcohol?<br>• Do you cook by yourself? Are there any changes with time spent on cooking?<br><br>**[Impact on work and housework]**<br><br>[Only for those who are working] I'm going to ask about your work.<br><br>• What do you do?<br>➢ Is it physical labor?<br>➢ Does your job require concentration?<br>➢ Do you often interact with people?<br>➢ Work style (full-time or part-time) *Confirm occupation from the demographic information above<br>➢ What are your daily work hours?<br>• Has there been any impact on your work since you started coughing?<br>➢ Have you ever had to quit your job due to coughing or experience a change in position (e.g., from sales to office work)?<br>➢ How many hours have you been absent from work due to health issues? Please count all cases when you were absent from work, late for work and left early due to illness.<br>➢ Has there been any changes in the amount of work<br>➢ How much have your health issues impacted your productivity?<br>➢ What is the cause? (e.g., physical or mental problems)<br><br>I'm going to ask about impact on housework.<br>• What kind of housework do you do?<br>➢ If not mentioned, ask about "cleaning," "laundry," "cooking," "gardening," and going out for shopping or banking, etc.<br>• How many hours do you spend on housework?<br>• How much do you feel your coughing symptoms impacts day-to-day housework?<br>➢ What do you think are the causes?

Time	Item
	**[Impact on family, friends, and communications]**<br><br>Now, I'm going to ask about its impact on relationships with friends and family.<br><br>• Has there been any changes in the relationship with your friends since coughing started?<br>➢ Range of interaction with people<br>➢ Frequency of meeting friends or acquaintances<br>➢ Behaviors you have avoided or have stopped in your relationships with family or friends (i.e., going out for dinner)<br>➢ Interest or sympathy for others: Do you feel more irritated toward others?<br>• What do you think are the reasons for changes in the relationship with friends?<br>• Have you changed your attitude towards your family?<br>➢ Can you express your feelings to your family, as you did before?<br>➢ Any changes with expression of love to your family<br>➢ Any changes with the relationship with your spouse<br><br>Now, I'll ask about impact on communication.<br>• Has there been any changes in your attitude, language or content when speaking to someone in-person since coughing started?<br>• Are there any changes with gesturing during talking?<br>• Are there any changes with the frequency, time and content of phone calls?<br>• Has there been any changes with the contents and expressions in your emails?<br>• Has there been any changes with your understanding of others?<br>• Has there been any changes with other people's behavior towards you or their understanding about you? (e.g., feel more distant than before)<br><br>**[Impact on leisure and hobbies]**<br><br>Now, I'm going to ask about your leisure and hobbies.<br>• Has there been any changes in the time spent on leisure activities since coughing started?<br>➢ Do you have any hobbies?<br>➢ Has there been any changes with the time spent on your hobbies?<br>➢ Has there been any changes with the frequency and the time spent to go out?

<table>
<tr><th>Time</th><th>Item</th></tr>
<tr><td></td><td>

Has there been any changes with time spent watching TV or reading books?
Has there been any behaviors that you stopped after you having coughing symptoms? (e.g., watching movies)

[Impact on economic burden and burden on hospital visit]
Now, I will ask about economic burden.

Do you feel burdened with the cost of treating your cough?
How about the indirect cost, such as the transportation fee for visiting the hospital.
Is your medical institution easily accessible for you?

Is there anything else you would want to add?
</td></tr>
</table>

*The original version of the interview guide was provided in Japanese
